# Aurora kinase A suppresses metabolic stress-induced autophagic cell death by activating mTOR signaling in breast cancer cells

**DOI:** 10.18632/oncotarget.2241

**Published:** 2014-07-22

**Authors:** Ling-Zhi Xu, Zi-Jie Long, Fei Peng, Yang Liu, Jie Xu, Chang Wang, Lei Jiang, Tao Guo, Muhammad Kamran, Si-Si Li, Chun-Li Wang, Hong-Jiang Wang, Yong-Fu Zhao, Xian-Yao Wan, Quentin Liu

**Affiliations:** ^1^ Institute of Cancer Stem Cell, Dalian Medical University, Dalian, China; State Key Laboratory of Oncology in South China, Cancer Center, Sun Yat-sen University, Guangzhou, China; ^2^ Department of Hematology, the Third Affiliated Hospital, Sun Yat-sen University, Guangzhou, China; ^3^ Institute of Hematology, Sun Yat-sen University, Guangzhou, China; ^4^ Department of Breast Surgery, the First Affiliated Hospital, Dalian Medical University, Dalian, China; ^5^ Department of Thyroid Surgery, the Second Affiliated Hospital, Dalian Medical University, Dalian, China; ^6^ Department of Critical Care Medicine, the First Affiliated Hospital, Dalian Medical University, Dalian, China

**Keywords:** aurora kinase, metabolic stress, autophagy, cell death, breast cancer

## Abstract

Aberrant Aur-A signaling is associated with tumor malignant behaviors. However, its involvement in tumor metabolic stress is not fully elucidated. In the present study, prolonged nutrient deprivation was conducted into breast cancer cells to mimic metabolic stress in tumors. In these cells, autophagy was induced, leading to caspase-independent cell death, which was blocked by either targeted knockdown of autophagic gene ATG5 or autophagy inhibitor 3-Methyladenine (3-MA). Aur-A overexpression mediated resistance to autophagic cell death and promoted breast cancer cells survival when exposed to metabolic stress. Moreover, we provided evidence that Aur-A suppressed autophagy in a kinase-dependent manner. Furthermore, we revealed that Aur-A overexpression enhanced the mammalian target of rapamycin (mTOR) activity under metabolic stress by inhibiting glycogen synthase kinase 3β (GSK3β). Inhibition of mTOR activity by rapamycin sensitized Aur-A-overexpressed breast cancer cells to metabolic stress-induced cell death. Consistently, we presented an inverse correlation between Aur-A expression (high) and autophagic levels (low) in clinical breast cancer samples. In conclusion, our data provided a novel insight into the cyto-protective role of Aur-A against metabolic stress by suppressing autophagic cell death, which might help to develop alternative cell death avenues for breast cancer therapy.

## INTRODUCTION

As an evolutionarily conserved catalytic process, autophagy is essential for maintaining and promoting cellular homeostasis and genome stability [1-4]. During autophagy, damaged cellular organelles and long-lived proteins are sequestered into double-membraned autophagosomes and delivered to lysosomes for degradation and recycling [2, 5]. Different types of cellular stress can trigger induction of autophagy, such as metabolic stress, a hall marker of tumor microenvironment [6]. Autophagy helps to limit cellular damage and maintain survival in response to metabolic stress [7, 8]. However, increasing lines of evidence indicate autophagy as an alternative death type under persistent stress conditions. Unlike apoptosis, autophagic cell death is morphologically described and occurs with massive autophagic vacuoles formation, lacking nuclear fragmentation, membrane blebbing and caspase activation [9, 10]. For example, prolonged oxidative stress generated by hydrogen peroxide and 2-methoxyestradiol caused autophagic cell death independent of apoptosis in transformed and cancer cells [11]. Impairment of autophagy strongly protected MEFs against cell death after prolonged nutrient deprivation in a PARP-1-dependent manner [12]. Moreover, chemical agents, such as arsenic trioxide, induced autophagic cell death in malignant glioma cells by upregulating BNIP3 [13]. Autophagy synergistically enhanced cell death when combining the ErbB1/2 inhibitor lapatinib and the BCL-2 family inhibitor obatoclax in colon and breast cancers [14]. Thus, the double-edged sword function of autophagy is controversial. Understanding the involvement of autophagy within metabolic stress is of great interest to develop therapeutic avenues for cancer treatment.

Aurora kinase A (Aur-A), which belongs to the mitotic Aurora serine/threonine kinase family, is essential for proper timing of mitotic entry and formation of bipolar spindles [15, 16]. We and other investigators found that Aur-A was overexpressed in numerous cancer types, such as laryngeal, colon, breast cancer and leukemia [17-20]. Dysregulation of Aur-A was associated with tumor metastasis and therapeutic resistance [21, 22]. In addition, our recent work revealed that kinase targeted inhibition of Aur-A caused polyploidization and elevated glycolytic metabolism level [23]. Kinase inhibition of Aur-A triggered induction of autophagy in breast cancer and leukemia cells [23, 24]. Both findings broadened the role of Aur-A in autophagy and cancer metabolic stress. However, the detailed mechanism has not been well understood, and the biological effects remain to be further explored.

Here, we set out to investigate the involvement of Aur-A within breast cancer cells undergoing persistent metabolic stress. We found that in response to prolonged nutrient deprivation, autophagy was induced and displayed as a cellular death mechanism, which was suppressed by Aur-A overexpression. Besides, we reported that GSK3β/mTOR signaling cascade was required for suppression of autophagy by Aur-A. Furthermore, analysis of clinical breast cancer samples revealed that Aur-A expression was negatively correlated with autophagic levels. All of these findings indicated a novel defense mechanism of Aur-A against metabolic stress in breast cancer.

## RESULTS

### Persistent nutrient deprivation induced-autophagy is suppressed by Aur-A

**Figure 1:**
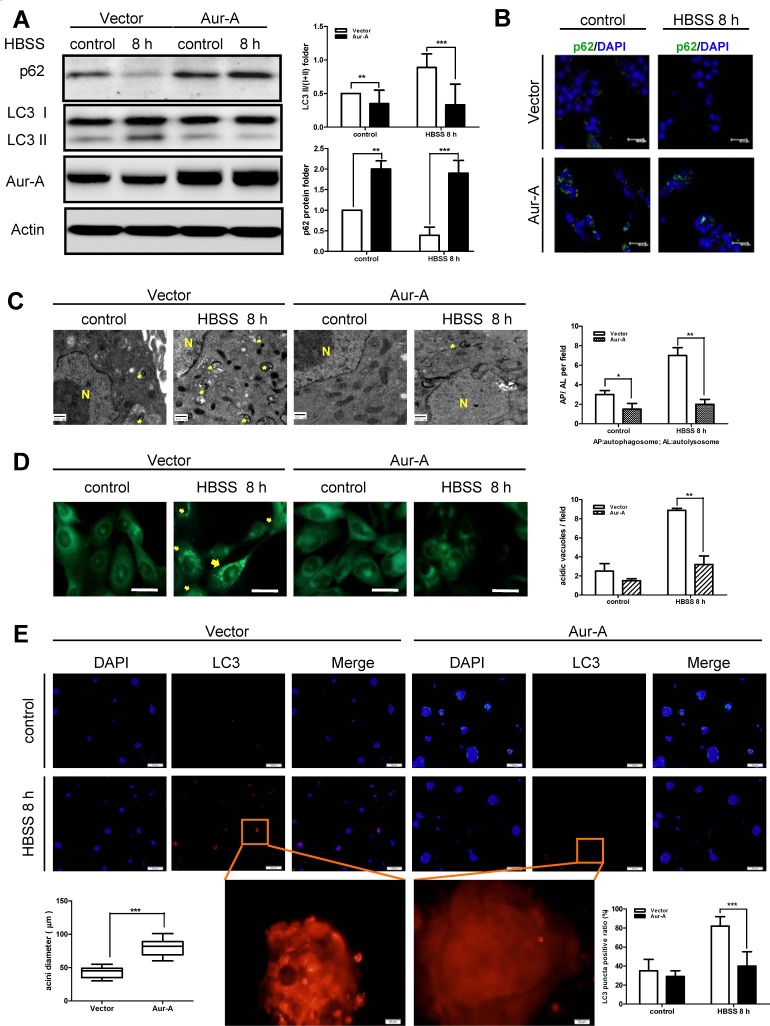
HBSS starvation induced-autophagy was suppressed by Aur-A overexpression Both control and Aur-A overexpressed cells were cultured in petri dishes and subjected to HBSS treatment for indicated time. After starvation, autophagic features were assessed by Western blot (A, SKBR-3 cells), immunofluorescence staining of p62 (B, SKBR-3 cells), transmission electron microscopy (C, SKBR-3 cells) (autophagosome or autolysosome, arrow indicated; N: nucleus) and MDC staining (D, BT-549 cells, scale length = 20μm). E, Both control and Aur-A overexpressed SKBR-3 cells stably expressing LC3-DsRed were cultured in three-dimensional system. Tumor masses were subjected HBSS starvation on day 14 and LC3-DsRed punctas were observed under a fluorescence microscopy. Nucleuses were visualized by DAPI staining. (A) Columns were averaged from three independent Western blot analysis; (C)~(E) Graphs were statistically analyzed from five random fields. (*p<0.05, **p<0.01, ***p<0.001)

In order to mimic metabolic stress in established tumors, we subjected breast cancer cells to prolonged Hank's buffer salt solution (HBSS) starvation (at least 8 hours). Nutrient deprivation significantly increased ROS generation in both Aur-A-overexpressed and control SKBR-3 cells (Supplementary Fig. S1). During metabolic stress, the production of ROS played an important role in triggering autophagy [6, 7]. Western blot analysis showed that autophagy was significantly induced after starvation in control SKBR-3 cells, as indicated by increased LC3 conversion, a marker of autophagy induction, and p62 degradation [25]. However, the induction of autophagy was suppressed when Aur-A was ectopically overexpressed (Fig. 1A). Transient knockdown of Aur-A by siRNA promoted HBSS starvation-induced autophagic features (Supplementary Fig. S2). Consistently, immunofluorescence staining revealed much more p62 protein within cytoplasm in Aur-A overexpressed SKBR-3 cells under both normal and starvation conditions, indicating a blockage of autophagy (Fig. 1B). Ultrastructural analysis by transmission electron microscope showed that autophagosomes with double membranes and autolysosomes containing cellular debris were detected in control SKBR-3 cells after starvation, which were rarely observed when Aur-A was overexpressed (Fig. 1C, arrow indicated). Similarly, the formation of autophagic vacuoles was suppressed by Aur-A after starvation in BT-549 cells, as detected by monodansylcadaverine (MDC) labeling (Fig. 1D, arrow indicated).

To further corroborate the above finding, Aur-A-overexpressed and control SKBR-3 cells, both of which stably expressed LC3-DsRed fusion protein, were cultured in 3D morphogenesis system. After tumor mass formation, nutrient deprivation was performed by HBSS treatment. Control cells showed obvious LC3-DsRed punctuate aggregates after subjected to starvation. In contrast, Aur-A overexpression attenuated the above phenomenon (Fig. 1E). These data further indicated that Aur-A suppressed autophagy in response to nutrient deprivation.

### Autophagy acts as a death mechanism during persistent metabolic stress

**Figure 2:**
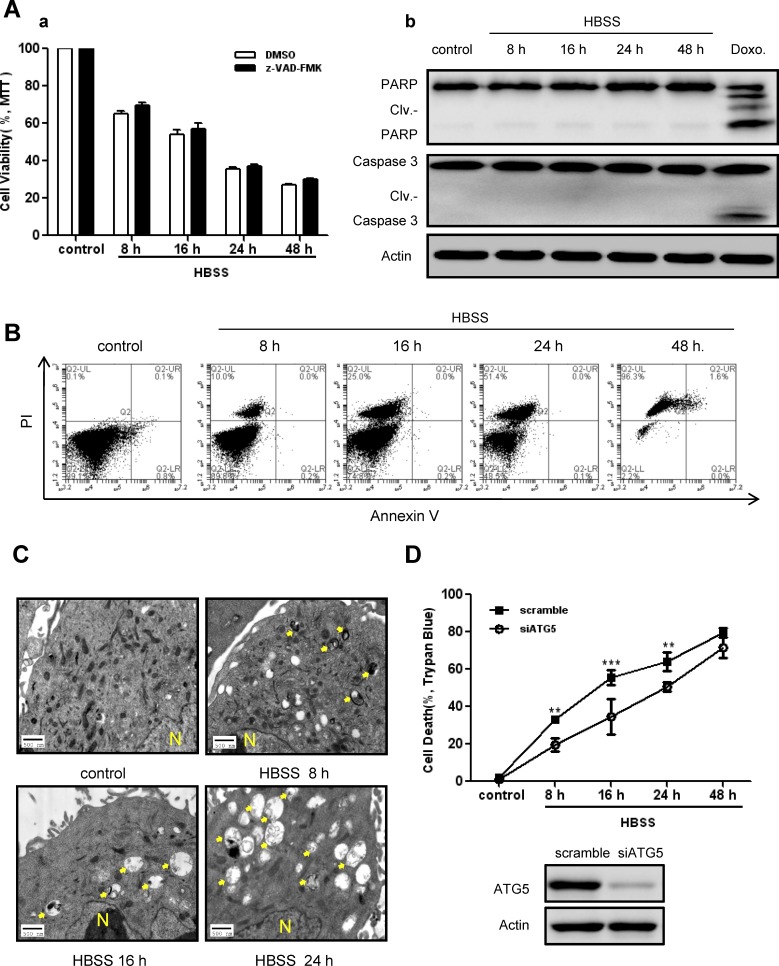
Autophagy acted as a death mechanism during persistent metabolic stress A, SKBR-3 cells were subjected to prolonged HBSS starvation (8 hours to 48 hours) with or without pretreatment by pan-caspase inhibitor z-VAD-FMK. Cell viabilities were measured by MTT assay (a). Data were mean ± S.D. of three independent experiments done in parallel. Caspase activation features were assessed by Western blot analysis (b). Doxorubicin (1μg/ml) treatment was used as a positive control. B, Apoptotic ratio was measured by Annexin-V/PI staining using flow cytometry in BT-549 cells after HBSS treatment. C, Autophagic vacuoles in SKBR-3 cells during prolonged starvation were detected by TEM (arrow indicated; N: nucleus). D, SKBR-3 cells were transfected with siATG5 or scramble sequence 24 hours before subjected to prolonged starvation. Cell death ratios were measured by trypan blue assay. Data were mean ± S.D. of three independent experiments done in parallel (^**^p<0.01, ^***^p<0.001). siRNA knockdown efficiency was assessed by Western blot.

A common cellular response to metabolic stress is cell death. As shown in Figure 2A (a, left panel), exposure to prolonged nutrient deprivation caused loss of cell viability in SKBR-3 cells. In order to determine the predominant death type in response to prolonged nutrient deprivation, both apoptosis and autophagic features were assessed. Cell viability showed no significant changes in the presence of z-VAD-FMK, a pan-caspase inhibitor (Fig. 2A-a), suggesting the cellular death might not depend on caspase activation. This finding was confirmed by Western blot analysis which showed no significant cleavage of PARP and Caspase-3 (Fig. 2A-b). Similarly, apoptosis detection by Annexin-V/PI using flow cytometry showed limited apoptotic features (Fig. 2B). In contrast, ultrastructural analysis showed the number of autophagy vesicles increased in a starvation time-dependent manner (Fig. 2C). These data raised the possibility that autophagy might be related to cell death under persistent metabolic stress in breast cancer cells. To further verify our hypothesis, autophagy was genetically inhibited by targeted knockdown of autophagic gene ATG5 in SKBR-3 cells prior to HBSS treatment. As expected, inhibition of autophagy increased cell viability at each time course (Fig. 2D). Consistently, pharmacological inhibition of autophagy by 3-Methyladenine (3-MA) pretreatment rescued cell death in BT-549 cells in response to HBSS starvation (Supplementary Fig. S3). All of these findings suggested that autophagy acted as a death mechanism during persistent metabolic stress in breast cancer cells.

### Aur-A protects breast cancer cells against metabolic stress by suppressing autophagy

**Figure 3:**
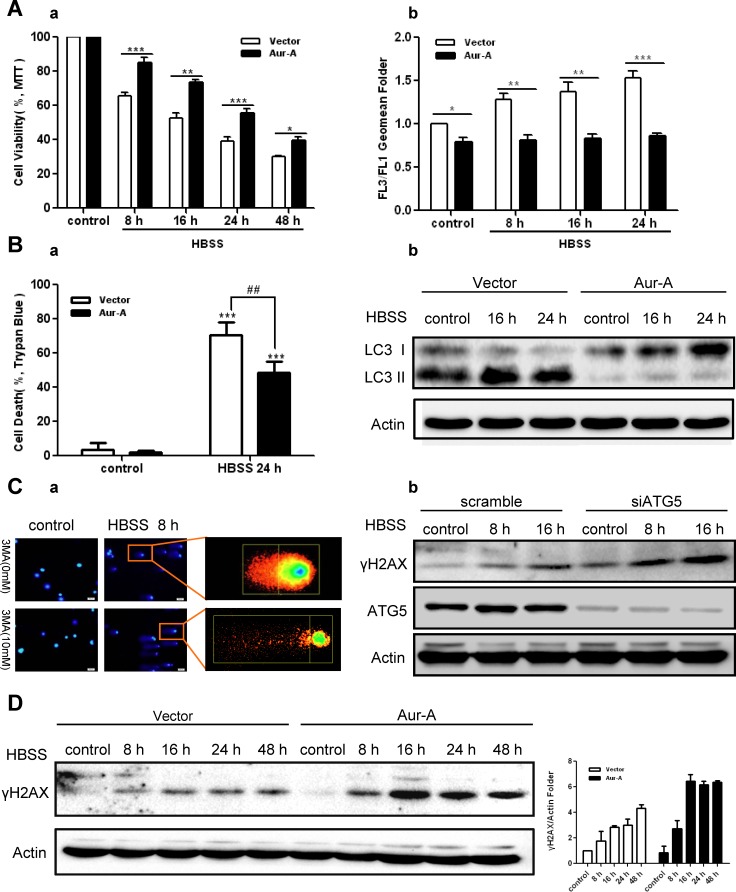
Aur-A protected breast cancer cells against metabolic stress by suppressing autophagy A, Both control and Aur-A overexpressed SKBR-3 cells were conducted to prolonged starvation. Cell viabilities were measured by MTT assay (a). Autophagic vacuoles were assessed by AO staining using flow cytometry, and autophagic levels were determined by a ratio between FL3 and FL1 geomean intensity (b). Data were mean ± S.D. of three independent experiments done in parallel (^*^p<0.05, ^**^p<0.01, ^***^p<0.001). B, Both control and Aur-A overexpressed BT-549 cells were treated with the same condition as (A). Cell viabilities were measured by trypan blue assay (a). Data were mean ± S.D. of three independent experiments done in parallel (^##^*p* & ^**^p<0.01, ^***^p<0.001). Autophagic levels were detected by Western blot analysis (b). C, Autophagy was inhibited either by 3-MA (a, BT-549 cells) or by siATG5 knockdown (b, SKBR-3 cells). DNA damage accumulations were measured either by Comet assay (a) or Western blot analysis of γH2AX phosphorylation levels (b). D, γH2AX phosphorylation levels were analyzed by Western blot in both control and Aur-A overexpressed SKBR-3 cells after prolonged starvation. Column graphs were averaged from three independent experiments and representative results were shown.

To explore the biological effect of autophagy suppression by Aur-A, we subjected Aur-A-overexpressed and control breast cancer cells to prolonged nutrient deprivation (as long as 48 hours). Time course analysis showed that ectopic Aur-A overexpression prevented loss of cell viability in both SKBR-3 and BT-549 cells, accompanied with decreased autophagic levels (Fig. 3A-B). These data indicated that Aur-A protected breast cancer cells against metabolic stress by suppressing autophagic cell death. Autophagy was essential for maintaining genome stabilities by eliminating damaged organelles and species [26, 27]. Consistent with previous findings, COMET assay and γH2AX signals showed that DNA damage was aggravated by inhibition of autophagy in face of stress (Fig. 3C). Ectopic Aur-A overexpression exacerbated γH2AX signals accumulation in both SKBR-3 and BT-549 cells (Fig. 3D & Supplementary Fig. S4), suggesting that by suppressing autophagic cell death, Aur-A might promote tumor progression under metabolic stress in breast cancer cells.

### Kinase activity of Aur-A contributes to suppression of autophagy

**Figure 4:**
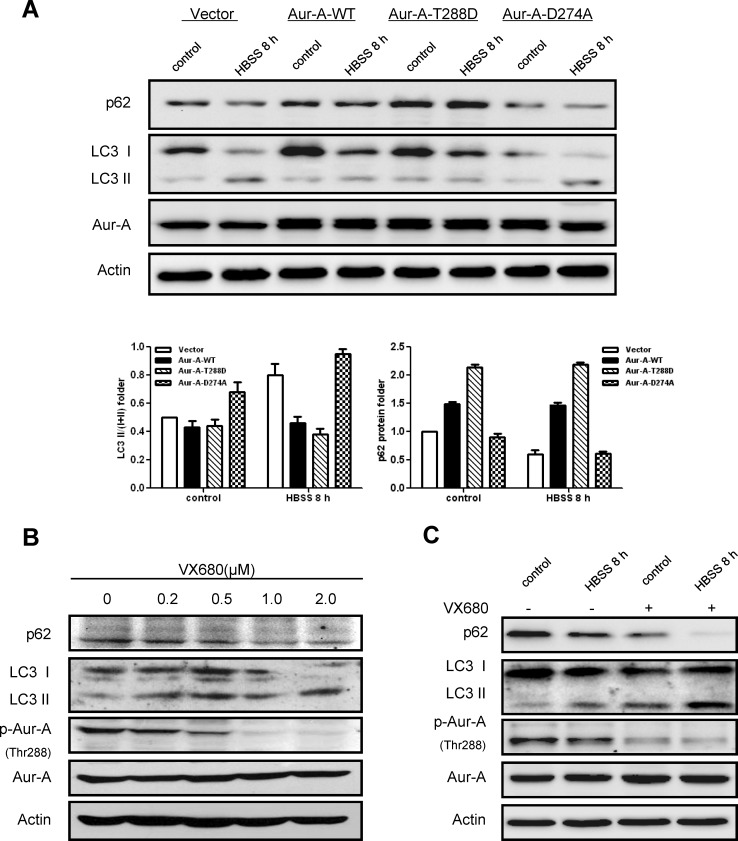
Kinase activity of Aur-A contributed to the suppression of starvation induced-autophagy A, Plasmids encoding different kinase forms of Aur-A were introduced into MCF-10A cells and autophagic levels were assessed by Western blot. Column results were averaged from triplicate experiments. B, SKBR-3 cells were treated with different dosages of VX680. Autophagic levels were analyzed by Western blot. C, SKBR-3 cells were conducted to HBSS starvation with or without VX680 (1μM) pretreatment for 12 hours. Autophagic levels were analyzed by Western blot.

As a serine/threonine kinase, Aur-A acts mainly via its kinase activity [15]. Thus we asked whether kinase activity of Aur-A participated in the suppression of autophagy under metabolic stress. MCF-10A cell, an immortalized breast epithelial cell line, was transfected with plasmids encoding different forms of Aur-A. Western blot analysis showed less LC3 conversion and p62 degradation were detected when wild type Aur-A (Aur-A-WT) or continuously activated Aur-A (Aur-A-T288D) were ectopically overexpressed. By contrast, forced overexpression of kinase dead form of Aur-A (Aur-A-D274A) attenuated the changes above (Fig. 4A). VX680, a kinase inhibitor of Aur-A, caused a dose-dependent induction of autophagy in SKBR-3 cell (Fig. 4B). Moreover, SKBR-3 cell pretreated with VX680 markedly sensitized to nutrient deprivation-induced autophagy, as measured by elevated conversion of LC3 and p62 degradation (Fig. 4C). Thus, these data revealed that kinase activity of Aur-A contributed to the suppression of nutrient deprivation-induced autophagy.

### Aur-A activates mTOR activity by inhibiting GSK3β under metabolic stress

**Figure 5:**
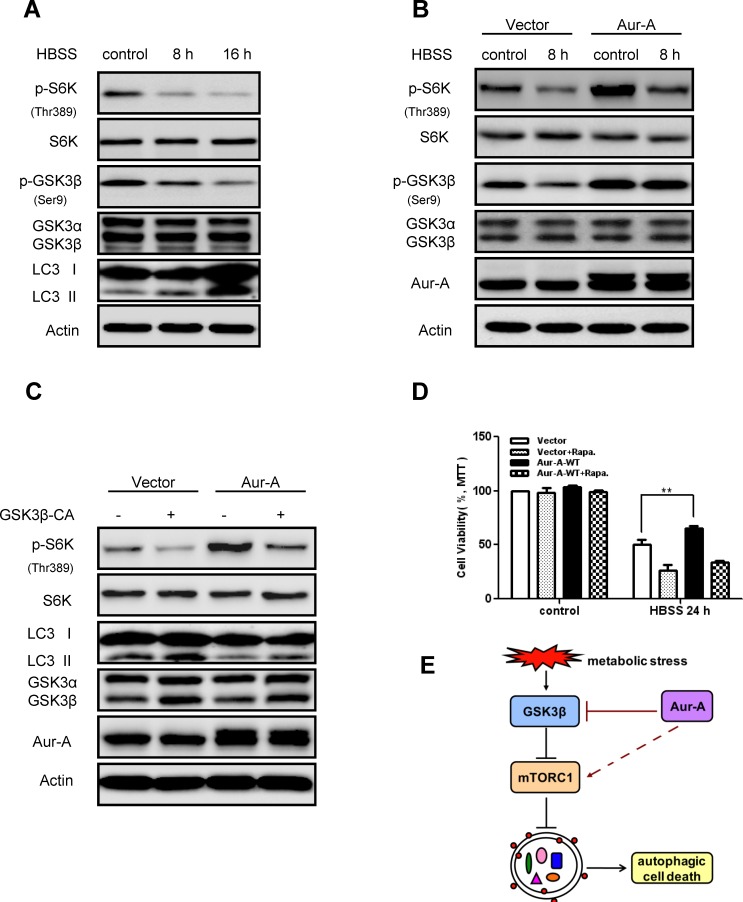
Aur-A activated mTOR signaling by inhibiting GSK3β under metabolic stress A-B, mTOR activity (indicated by p70S6K phospho-Thr389 level) and GSK3β activity (indicated by phospho-Ser9 level) were assessed by Western blot after HBSS starvation in control and Aur-A overexpressed SKBR-3 cells. C, GSK3β activity was reconstituated by transfection of V5-GSK3β-CA plasmid in control and Aur-A overexpressed SKBR-3 cells. After HBSS starvation, mTOR activity (indicated by p70S6K phospho-Thr389 level) and LC3 conversion (LC3 II/total) were assessed by Western blot. D, Cells were pretreated with rapamycin 12 hours before subjected to HBSS treatment. After starvation, cell viabilities were measured by MTT assay. Data were mean ± S.D. of three independent experiments done in parallel (^**^p<0.01). E, A working model to elucidate signaling pathway that mediated action of Aur-A under metabolic stress.

In an effort to dissect possible mechanisms whereby Aur-A suppressed autophagy, we focused on mTOR signaling, a key cellular nutrient and energy sensor, which is tightly regulated during metabolic stress [28, 29]. Western blot analysis showed that phosphorylation of p70S6 kinase (S6K) on Thr389, which is a direct mTOR phosphorylation site and positively correlates with mTOR activity, decreased along with nutrient deprivation in SKBR-3 cells (Fig. 5A), suggesting mTOR signaling was inhibited in response to metabolic stress. Forced expression of Aur-A enhanced phosphorylation of S6K on Thr389 under both control and nutrient deprivation conditions (Fig. 5B), indicating Aur-A might suppress autophagy by triggering mTOR activity under metabolic stress.

Recent studies strongly suggested that GSK3β inhibited mTOR signaling [30, 31] and GSK3β was activated in nutrient deprivation [31, 32]. Phosphorylation of GSK3β on Ser9, which is negatively correlated with its kinase activity, decreased in response to nutrient deprivation in SKBR-3 cells (Fig. 5A). Aur-A overexpression attenuated the changes above (Fig. 5B). Previous work revealed that Aur-A inhibited GSK3β activity by phosphorylating it on Ser9 [33, 34]. Pharmacological inhibition of GSK3β activity caused mTOR signaling activation and autophagy induction in both SKBR-3 and BT-549 cells (Supplementary Fig. S4). Thus it prompted us to speculate whether GSK3β inactivation was required for Aur-A to activate mTOR signaling in breast cancer. To test our hypothesis, we reconstituted GSK3β kinase activity by introducing either a control vector or continuously activated form of GSK3β (GSK3β-CA) into SKBR-3 cell. Western blot analysis showed that reconstitution of GSK3β activity, to a large extent, repressed mTOR activation by Aur-A overexpression, as well as increased LC3 conversion during nutrient deprivation (Fig. 5C). These data suggested GSK3β suppression was essential, at least in part, for Aur-A to activate mTOR signaling under metabolic stress. Besides, cell survival maintained by Aur-A overexpression in response to nutrient deprivation was restrained by suppression of mTOR activity (Fig. 5D), indicating a critical role of mTOR for the aberrant Aur-A signaling in breast cancer cells.

### Tumoral Aur-A expression is negatively correlated with autophagic levels in breast cancer samples

**Figure 6:**
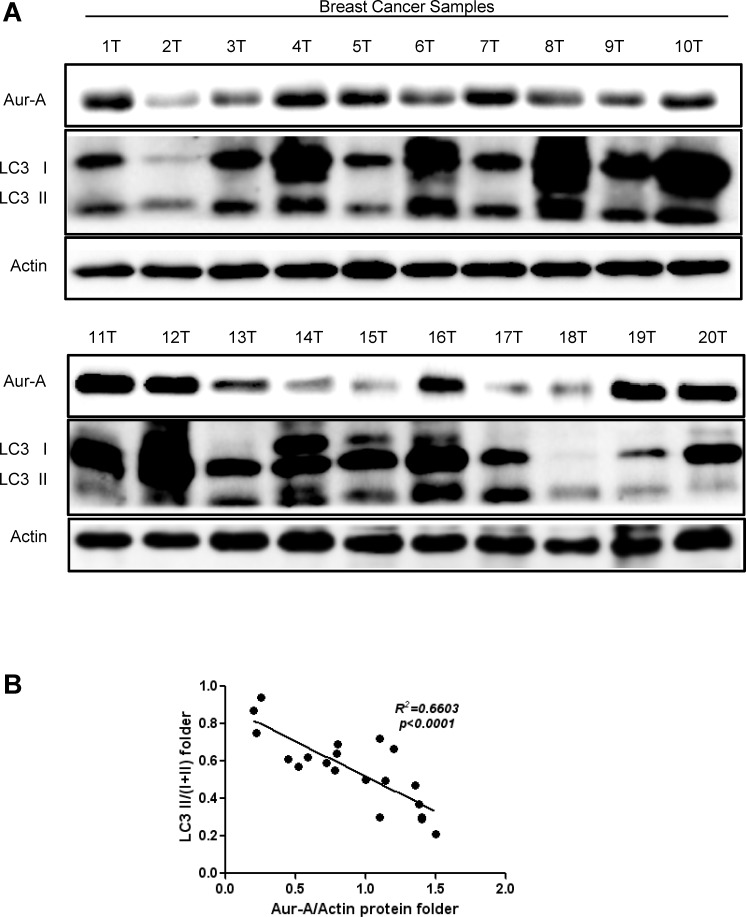
Aur-A expression was negatively correlated with autophagic level in breast cancer samples A, Breast cancer samples were lysed and subjected to Western blot to analyze Aur-A expression and LC3 conversion levels (indicated by LC3 II/total; n=20; T: tumor). B, Both Aur-A expression and LC3 conversion levels were normalized to T1 (a) and linear regression analysis was performed (b).

Finally, we assessed expression of Aur-A and autophagic level in human breast cancer samples by Western blot analysis. The conversion of LC3 I to LC3 II (LC3 II/I+II) was relatively lower in samples which showed much more abundant Aur-A expression (Fig. 6A). Linear regression analysis showed an inverse correlation between Aur-A expression and conversion of LC3 (Fig. 6B, *R^2^=0.6603, p<0.0001*), verifying that Aur-A overexpression was associated with low autophagic level in vivo.

## DISCUSSION

By conduction of prolonged nutrient deprivation into breast cancer cells, several novel findings were presented in the current study: (i) autophagy was induced in response to persistent metabolic stress, and functioned as a caspase-independent cellular death mechanism; (ii) Aur-A mediated resistance to persistent metabolic stress-induced cell death by suppressing autophagy in a kinase-dependent manner; (iii) Aur-A overexpression enhanced and prevented the complete loss of mTOR activity during metabolic stress, partially through inhibiting GSK3β; (iv) clinically, analysis of breast cancer samples further verified that the tumoral overexpression of Aur-A was correlated with lower autophagic level.

As a pathway for stress tolerance, autophagy occurs in response to metabolic stress, but conflicting evidence complicate the effect of autophagy on cell survival or death [1, 35]. One possible interpretation is that autophagy functions as a temporary survival mechanism for cellular viability under diverse stress conditions [7, 8]; however, if these conditions exist for a long time, excessive autophagy will render cells unable to cope with stress because of the destruction of cellular structures and energy exhaustion, leading to autophagic cell death [10, 36]. Tumor cells are frequently subjected to metabolic stress, arising from increased biosynthetic needs, inadequate vascularization, and shortage of nutrient supply [6]. Thus, we conducted prolonged HBSS nutrient deprivation (at least 8 hours) in breast cancer cell lines, which mimicked the simulation of persistent metabolic stress in established breast tumors in vivo. Under such circumstances, autophagy was induced (Fig. 1), and displayed as a cellular death mechanism (Fig. 2). Consistent with our findings, recent studies reported that persistent oxidative stress generated by hydrogen peroxide (H_2_O_2_) and 2-methoxyestradiol (2-ME) caused autophagic cell death independent of apoptosis in transformed and cancer cells [11]. Prolonged exposure to hypoxia induced autophagic cell death in apoptosis-competent solid tumor cells [37]. However, since inhibition of either apoptosis or autophagy did not block loss of cell viability totally (Fig. 2), it was in a possibility that multiple death pathways participated in cell death under metabolic stress. Further efforts are needed to well understand detailed death mechanisms involved in cancer metabolic stress.

As an onco-protein, the pro-tumor action of Aur-A has been widely documented via different mechanisms, such as promoting metastasis, drug resistance, genome instability and so on [15, 17, 21, 38]. Autophagic cell death, also referred as type II programmed cell death, was essential for maintaining genome stabilities and tumor suppression [26, 27, 39]. Our data showed that autophagic cell death helped to mitigate metabolic stress-induced genome damage, while Aur-A overexpression suppressed autophagic cell death, promoting DNA damage accumulation (Fig. 3C). Besides, in 3D culture system, which offers the possibility for cancer cells to resemble tumorigenesis by forming a compact tumor cell mass in vitro [40], nutrient deprivation induced-autophagy was suppressed by Aur-A overexpression as well (Fig. 1D). A recent study reported that autophagy antagonized specific aspects of oncogenic PI3K transformation, and autophagy defects strongly enhanced cell proliferation during 3D morphogenesis [41]. Consistently, in our morphogenesis model, acini sizes were larger in Aur-A-overexpressed cells, accompanied with defective autophagic features. Based on these results, we concluded that by mediating resistance to autophagic cell death, Aur-A protected breast cancer cells against metabolic stress, which might promote tumor progression.

The current concept of treatment for breast cancer focuses on induction of tumor cell apoptosis. However, tumors harboring defective apoptosis signaling often show poor responses to chemotherapy. Therefore, it is urgent to develop alternative death mechanisms for the effective management of tumor therapies. Growing evidence indicated that therapeutic induction of autophagic cell death showed potential clinical benefits for cancer treatment. Arsenic trioxide induced autophagic cell death in malignant glioma cells by upregulating BNIP3 [13]. Obatoclax, the BCL-2 family inhibitor, promoted autophagy to enhance cell death when combining the ErbB1/2 inhibitor lapatinib in colon and breast cancer [14]. Our present data raised a novel possibility that targeting Aur-A might be expected to trigger autophagic cell death in apoptosis-defective tumors. VX680, a small aurora kinase inhibitor, was reported to mediate a synthetic lethal interaction with Myc by combining apoptosis and lethal autophagy [42]. In that report, initial lethal response (by the end of 3 days treatment) to VX680 was associated with activation of the canonical apoptosis; after 3 days exposure, autophagy was a major effecter of delayed death. Paradoxically, a previous work from our group showed that autophagy activation was responsible for resistance to VX680-induced apoptosis in breast cancer cells [24]. One possible explanation for the contradictory was the different exposure time and different cancer types. We exposed breast cancer cells to VX680 by 24 hours, during which autophagy might be activated as an early response to therapeutic stress [24]. Ongoing projects are trying to explore conditions that distinguish between survival-supporting and death-promoting effect of autophagy regulated by Aur-A under different stress conditions. Meanwhile, similar to our previous work [24], one recent study reported that Ras inhibitor Salirasib induced autophagy, which partially protected cancer cells from death [43]. Regardless of cellular backgrounds, it prompted us to ask whether increased autophagy during cancer therapy was a common sign of drug responsiveness (such as Aur-A and Ras targeted inhibitors) or resistance? In fact, inhibitors of autophagy could produce different outcomes: cell survival or cell death [44]. Thus, it is urgent for clinical physicians and cancer researchers to elucidate how to manipulate autophagy to improve cancer therapeutics.

As a central integrator of cell metabolism, growth, proliferation and survival, aberrant mTOR signaling participates in tumor autophagic responses to various stresses. Constitutive mTOR activation impaired autophagy in response to amino acid starvation and hypoxia [45]. Inhibition of mTOR was crucial for nutrient stress-induced autophagy via activating PIK3C3/VPS34 complexes [46]. Recently, clinical trials of combination chemotherapy and mTOR inhibitors in breast cancer demonstrated valuable results [47]. In this study, our data revealed that Aur-A mediated resistance to autophagic cell death through triggering mTOR activity under metabolic stress, suggesting a pro-survival role of mTOR for cancer cells undergoing metabolic stress, which indicated potential therapeutic benefits of mTOR inhibitors for breast cancer with activated Aur-A signaling. Conversely, a current model pointed out that in the combined context of growth factor scarcity and excessive nutrient, mTOR activation was triggered and detrimental to HEK293-T Phoenix cell survival; inhibition of mTOR by its kinase inhibitors or nutrient withdrawal dramatically reduced cell mortality [48]. The paradox between this model and our data might arise, to a large extent, from cellular genetic backgrounds and microenvironments: mTOR activity is hyperactivated by deregulated upstream factors in numerous cancer types, to adapt hypermetabolic demands and nutrient deficiency stresses [49]; however, mTOR dependent circuitry of metabolic toxicity occurred under excessive nutrient conditions [48], a feature shared by diabetes and other ageing-related pathologies. Therefore, we favor the idea that mTOR might be a versatile multitasker which takes on cancer and other pathologies.

Moreover, another important finding in the current study was the suppression of GSK3β activity underlying the mechanism whereby Aur-A activated mTOR signaling in breast cancer cells (Fig. 5C). As a key component of the canonical Wnt pathway, GSK3β inhibited mTOR in a TSC2-dependent manner [30, 31] and its activation was essential to induce autophagy in response to nutrient stress [31, 32]. Previously, we and others showed that Aur-A phosphorylated GSK3β at Ser9, thus inhibiting its kinase activity [33, 34]. Here we showed that reconstitution of GSK3β kinase activity repressed mTOR signaling with the presence of Aur-A overexpression. However, reconstitution of GSK3β activity did not totally attenuate mTOR activation by Aur-A (Fig. 5C), suggesting Aur-A might regulate mTOR signaling cascade by other mechanisms besides suppressing GSK3β activity.

Taken together, we demonstrated that kinase activity of Aur-A mediated a novel pro-survival mechanism under metabolic stress by suppressing autophagic cell death in breast cancer cells, which in turn might accelerate tumor progression. More importantly, our data indicated a potential therapeutic utilization of Aur-A kinase inhibitors to trigger alternative autophagic cell death in defective apoptosis breast cancer cells, which might shed light on tumor intervention and therapy.

## METHODS

### Clinical samples, cell lines and reagents

All breast cancer samples were obtained from newly diagnosed patients with prior patient consent and the approval of the Institutional Clinical Ethics Review Board in the 1^st^ Affiliated Hospital of Dalian Medical University. Samples were kept in liquid nitrogen for protein extraction.

HEK293T cell and human breast cancer cells (SKBR-3, BT-549) were cultured in DMEM medium (Invitrogen) or RMPI 1640 medium (Invitrogen) supplemented with 10% (v/v) fetal bovine serum (FBS, HyClone). The immortalized breast epithelial cell MCF-10A was cultured in DMEM/F12 medium (Invitrogen) supplemented with 5% (v/v) horse serum (HS, HyClone), 20ng/ml EGF, 100ng/ml cholera toxin, 0.01mg/ml insulin and 500ng/ml hydrocortisone. All cell lines were purchased from American Type Culture Collection (ATCC) and incubated at 37°C in humidified 5% CO_2_ incubator.

Reagents used were purchased as followed: VX680 (Selleck Chemicals, 639089-54-6), 3-Methyladenine (3-MA, Sigma, M9281), rapamycin (Sigma, 37094), SB216763 (Sigma, S3442), z-VAD-FMK (Beyotime, C1202), lithium chloride (LiCl, Sangon Biotech, LDB0307), doxorubicin (KeyGEN BioTECH, KGA8182). Nutrient deprivation was performed using Hank's balanced salt solution (HBSS, HyClone). To avoid any supplementary stress, HBSS was preheated at 37°C before added into cells.

### Three- dimensional culture

Three-dimensional culture was performed as previously described [40]. Briefly, culture slides (BD BioCoat^TM^) were coated with 50μl growth factor reduced Matrigel^TM^ (BD Biosciences) per well. Cells (1×10^3^) mixed with 2% Matrigel^TM^ were added to each well and refed every 3 days. Then cell masses were fixed, stained with DAPI (1μg/μl) and observed under a fluorescence microscope (Olympus).

### Immunofluorescence staining

Immunofluorescence staining of cells was done as previously described [17]. Briefly, cells were fixed in 2% para-formaldehyde-PBS at room temperature for 20 minutes and permeabilized in 0.5% Triton X-100 in PBS for 10 minutes at 4°C. Cells were then blocked with 1% BSA and incubated with primary antibody against p62 (Santa Cruz, sc-28359), followed by a FITC conjugated second antibody (Invitrogen), counterstained with DAPI (1μg/μl), and visualized using a confocal microscope (Leica).

### Monodansylcadaverine (MDC) staining

Monodansylcadaverine (MDC, Sigma) was applied to stain autophagic vacuoles as previously described [23]. Briefly, cells were fixed in 2% para-formaldehyde-PBS at room temperature for 20 minutes and incubated with the auto-fluorescent dye MDC with a final concentration of 0.05mM for 10 minutes at 37°C. Cells were then viewed under a fluorescence microscope (Olympus).

### Acridine Orange (AO) staining

Autophagy quantification by AO staining using flow cytometry was performed as previously described [50]. Briefly, cells were stained with 1μM AO for 15 minutes at 37°C, trypsinized, washed, and re-suspended in phenol red-free medium (Invitrogen) supplemented with 10% fetal bovine serum (HyClone). After filtration, single cell solution was detected by flow cytometry (BD FACS Calibur) with 488nm excitation light. Autophagy was quantified as a ratio between geomean fluorescence intensity of red vs. green fluorescence (FL3/FL1).

### Determination of ROS levels

2′-7′-dichlorodihydrofluorescein diacetate (DCF-DA, Sigma) was used as molecular probes to detect ROS levels within cells. Briefly, cells were washed once with warm PBS and labeled by DCF-DA with a final concentration of 10μM for 20 minutes at 37°C in dark before subjected to HBSS starvation. After treatment, labeled cells were washed with warm PBS, trypsinized, and analyzed in a flow cytometer (BD Accuri C6).

### Transmission Electron Microscopy (TEM)

Cells were fixed with 2.5% glutaraldehyde in 0.1M phosphate buffer (pH 7.4), followed by 1% osmium tetroxide (OsO4). After dehydration, penetration and aggregation, thin sections were stained with uranyl acetate and lead citrate for observation under a JEM 2000 EX transmission electron microscope.

### Lentivirus packaging and generation of transfection cell lines

pLVX-LC3-DsRed lentivirus was packaged in HEK293T cell and viral particles were collected 48 hours post transfection. After infection, cells stably expressing LC3-DsRed were chosen by selection with 2μg/ml puromycin (Sigma).

Cell lines with vector and stably overexpressed Aur-A (HA tagged) were constructed as previously described [24]. Site-directed mutations were performed using TaKaRa MutanBEST Kit according to the manufacturer's instructions. V5-GSK3β-CA (continuously activated) and empty vectors were generously provided by Professor Tie-Bang Kang (Cancer Center, Sun Yat-sen University) and transiently transfected into cells prior to further analysis.

### COMET assay

The comet assay was performed under alkaline conditions as previously described [51]. Briefly, cells were mixed with low melting agarose in PBS and spread on slides. Cells were lysed for 1 hour at 4°C in a freshly prepared lysis buffer (pH 10) containing 2.5M NaCl, 100mM EDTA, 10mM Tris, 10% DMSO, and 1% Triton X-100. Slides were then placed for 20 minutes in freshly prepared electrophoresis buffer (300mM NaOH and 1mM EDTA, pH 10) at 4°C. Electrophoresis was carried out for 20 minutes at 25volts, 300mA. All these steps were carried out in the dark. Finally, the slides were rinsed in neutralization buffer (0.4M Tris-HCl, pH 7.5) for 5 minutes three times, stained with DAPI and observed with a fluorescence microscope.

### Small interfering RNA transfection

Transient transfection was performed by using Lipofectamine2000 (Invitrogen) according to the manufacturers' protocols. RNA oligonucleotide duplexes (Aur-A: 1-GGCUUUGGAAGACUUUGAATT, 2-CUGGCUCUUAAAGUGUUAUTT; ATG5: UGAUAUAGCGUGAAACAAGTT) were purchased from Shanghai GenePharma Co., Ltd.

### Cell viability detection

Cell viabilities were detected by MTT assay and trypan blue assay. (a) MTT assay: cells were seeded into 96-well flat bottom plates. After different treatment, 20μl of MTT solution (5mg/ml, Sigma) was added to each well and cells were incubated at 37°C for another 4 hours. The absorbance (OD) was measured at 492nm using a multimode plate reader (Perkin Elmer); (b) trypan blue assay: cells were seeded into 6-well flat bottom plates. After different treatment, cells were washed and trypsinized. Cell pellets were dissolved in trypan blue solution and the number of viable cells was counted under a normal microscope.

### Apoptosis detection

Detection of apoptosis was performed using the AnnexinV-FITC/propidium iodide (PI) Apoptosis Detection Kit (KGA107; Keygen). Briefly, cells were seeded in 6-well flat bottom. After treatment, cells were harvested, washed twice in phosphate buffer solution (PBS), and stained with AnnexinV-FITC/PI according to the manufacturer's instructions. The resulting fluorescence was detected by a flow cytometer (BD Accuri C6).

### Western blot analysis

Cellular or tissue samples were lysed with RIPA lysis buffer with freshly added cocktail protease inhibitor (Thermo Scientific). Equal amounts of cellular proteins were subjected to electrophoresis in SDS-PAGE, and transferred to nitrocellulose membranes (Millipore). The membranes were blocked and then incubated at 4°C overnight with indicated first antibodies, followed by incubation with appropriate HRP-conjugated secondary antibodies (Thermo Scientific). The protein bands were detected and analyzed with an ECL Western blotting detection kit (Avansta, K-12045-D50) using Bio-Rad ChemiDoc XRS+ Imaging System according to the manufacturer's instructions. First antibodies were purchased as follows: rabbit anti-LC3B (Sigma, L7543), rabbit anti-caspase 3 (Cell Signaling Technology, 9662), rabbit anti-PARP (Cell Signaling Technology, 9532), rabbit anti-phospho-Aurora A (Thr288) (Cell Signaling Technology, 3079), rabbit anti-phospho-S6K (Thr389) (Cell Signaling Technology, 9205), rabbit anti-ATG5 (Cell Signaling Technology, 2630), rabbit anti-phospho-GSK3α/β (Ser21/9) (Cell Signaling Technology, 9331), rabbit anti-β-Catenin (Millipore, 06-734), mouse anti-p62 (Santa Cruz, sc-28359), mouse anti-GSK3α/β (Santa Cruz, sc-7291), rabbit anti-S6K (Epitomics, 1494-1), rabbit anti-Aur-A (Upstate, 07-648), mouse anti-Actin (Proteintech, 60008-1-Ig), rabbit anti-phospho- GSK3β (Ser9) (Assaybiotech, A7098), rabbit anti-phospho-H2AX (Ser139) (ExCell Biology, IM001-0223).

### Statistical analysis

Data were expressed as mean ± SD of three independent experiments with GraphPad Prism software. Statistical analysis was performed using Statistical Package for Social Sciences (SPSS) software (version 16.0). The Student's *t*-test was used to make a statistical comparison between groups. *p* <0.05 was considered statistically significant.

## SUPPLEMENTARY MATERIAL FIGURES



## Abbreviations

3-MA: 3-methyladenine
AO: acridine orange
ATG: autophagy-related gene
Aur-A: aurora kinase A
DCF-DA: 2′-7′-dichlorodihydrofluorescein diacetate
DMSO: dimethyl sulfoxide
GSK3β: glycogen synthase kinase 3β
HBSS: Hank's balanced salt solution
LC3: microtubule-associated protein 1 light chain 3
LiCl: lithium chloride
MDC: monodansylcadaverine
mTOR: mammalian target of rapamycin
PARP: poly-ADP-ribose polymerase
ROS: reactive oxygen species
TEM: transmission electron microscopy
