# Supplementary material for: Aurora kinase A suppresses metabolic stress-induced autophagic cell death by activating mTOR signaling in breast cancer cells
Source: Oncotarget. 2014 Jul 22;5(17):7498–511. doi: 10.18632/oncotarget.2241 (PMC4202139; doi:10.18632/oncotarget.2241)
Supplement: Supplementary file 1 [file oncotarget-05-7498-s001.pdf]

Aurora kinase a suppresses metabolic stress-induced autophagic cell death by activating mTOR signaling in breast cancer cells

Supplementary Material

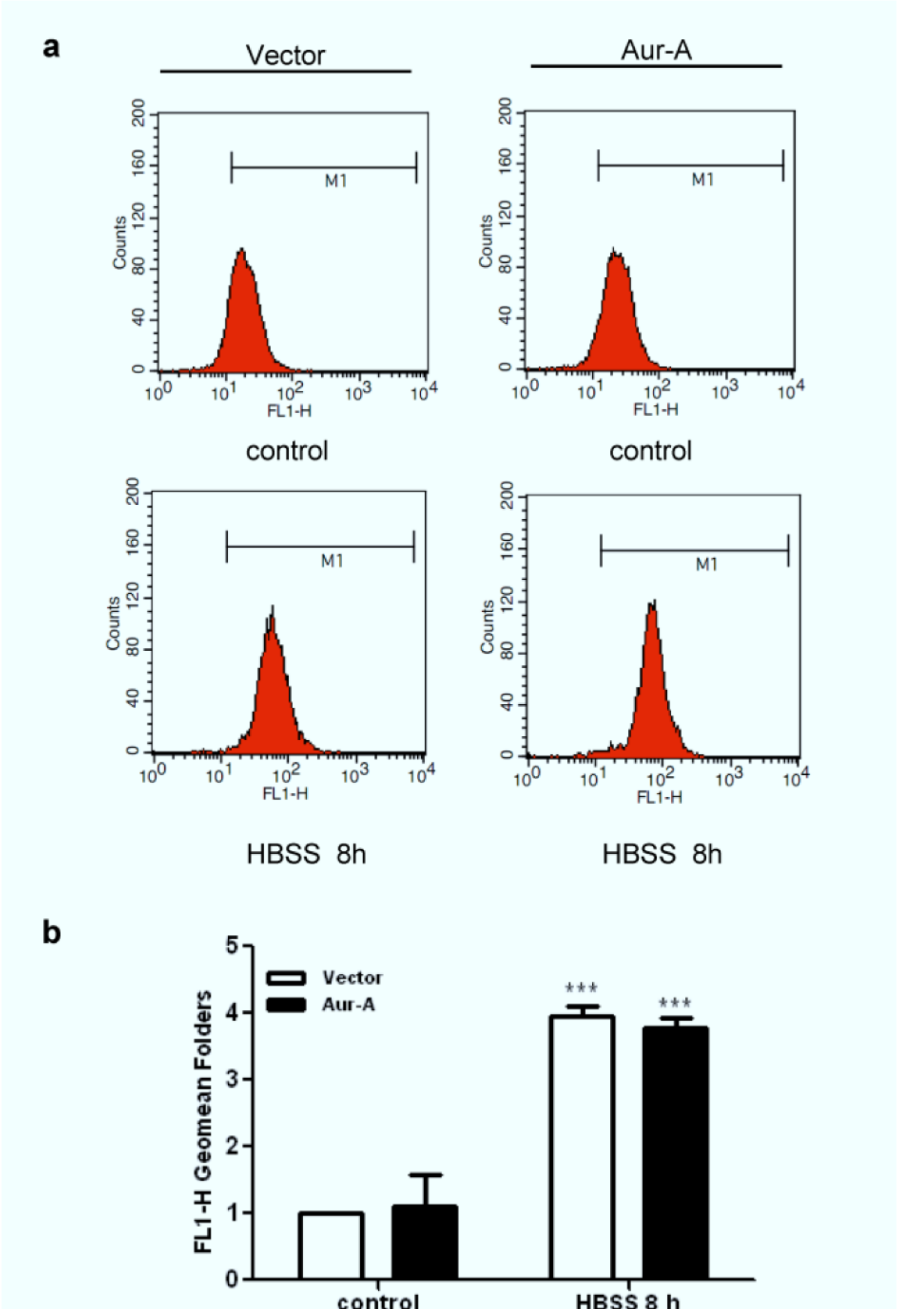

Supplementary Figure S1: ROS levels were increased by HBSS starvation in SKBR-3 cells. ROS levels were detected by DCF-DA labeling using flow cytometry in both control and Aur-A overexpressed SKBR-3 cells after HBSS treatment (a). Column graphs were averaged from three independent experiments (b; $^{***}p<0.001$).

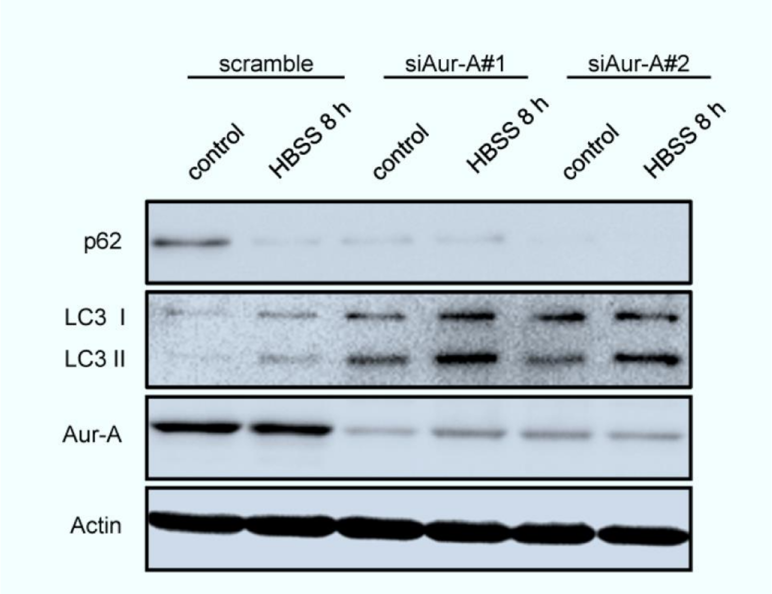

Supplementary Figure S2: Knockdown of Aur-A sensitized BT-549 cells to HBSS starvation-induced autophagy. Aur-A was transiently knocked-down by siRNA inteference 24 hours prior to HBSS starvation. After treatment, autophagic features were analyzed by Western blot, as indicated by LC3 conversion and p62 degradation.

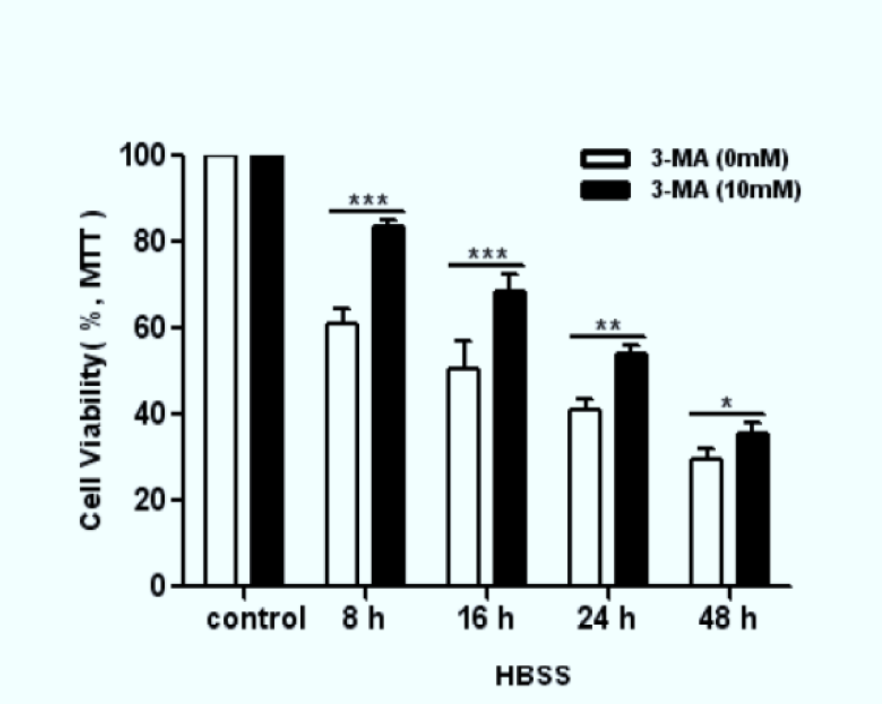

Supplementary Figure S3: Inhibition of autophagy rescued BT-549 cells from HBSS starvation-induced cell death. BT-549 cells were pre-treated with 3-MA before subjected to HBSS starvation. After treatment, cell viabilities were measured by MTT assay. Data were mean ± S.D. of three independent experiments done in parallel ($^{*}p$<0.05, $^{**}p$<0.01, $^{***}p$<0.001).

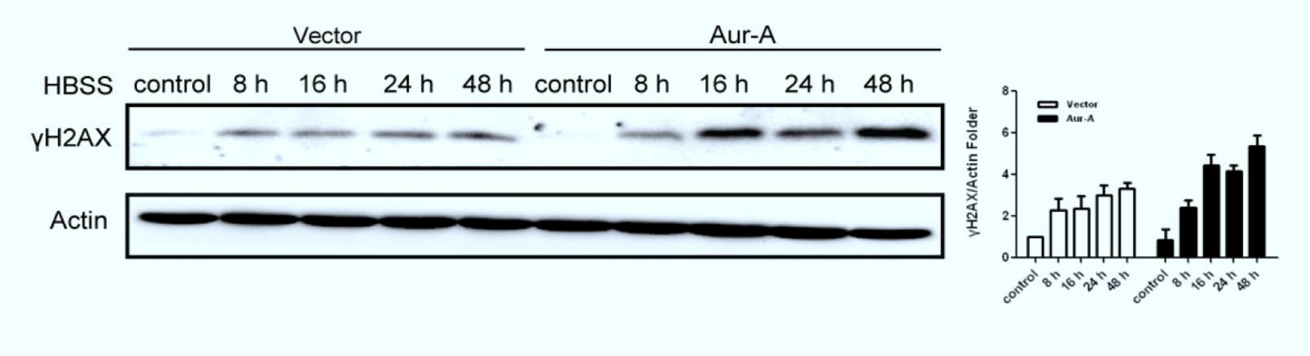

Supplementary Figure S4: Aur-A overexpression promoted HBSS starvation-induced DNA damage accumulation in BT-549 cells. γH2AX phosphorylation levels were analyzed by Western blot in both control and Aur-A overexpressed BT-549 cells after prolonged starvation. Column graphs were averaged from three independent experiments and representative results were shown.

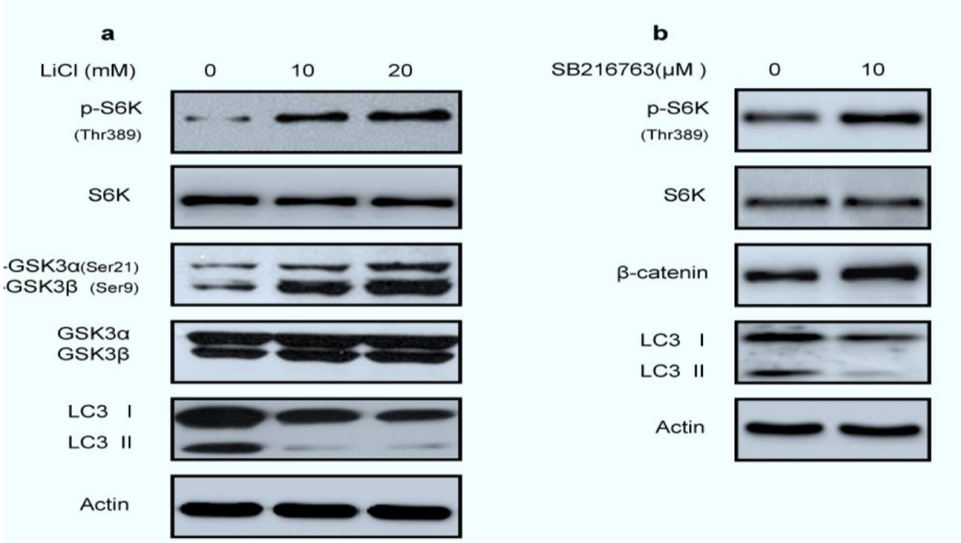

Supplementary Figure S5: Inhibition of GSK3 promoted mTOR activity in breast cancer cells. SKBR-3 (a) and BT-549 (b) cells were treated with LiCl and SB216763, respectively. mTOR activity (indicated by p70S6K phospho-Thr389 level) and autophagic levels (LC3 conversion) were assessed by Western blot analysis. Phospho-Ser21 of GSK3α and phospho-Ser9 of GSK3β were used to evaluate the drug efficiency of LiCl, and β-catenin for SB216763.
